# Agreement of ocular biometric measurements in young healthy eyes between IOLMaster 700 and OA-2000

**DOI:** 10.1038/s41598-020-59919-y

**Published:** 2020-02-21

**Authors:** Xuan Liao, Yue Peng, Bo Liu, Qing-Qing Tan, Chang-Jun Lan

**Affiliations:** 1 Department of Ophthalmology, Affiliated Hospital of North Sichuan Medical College, Nanchong, China; 2 Department of Ophthalmology and Optometry, North Sichuan Medical College, Nanchong, China

**Keywords:** Diagnostic markers, Lens diseases

## Abstract

This prospective cross-sectional study aimed to evaluate the agreement of two new biometers for measuring ocular biometric parameters in young healthy eyes. Ocular biometric parameters were measured using IOLMaster 700 and OA-2000. Power vector analyses of Cartesian (J0) and oblique (J45) components of corneal astigmatism were performed. The right eyes of 103 healthy volunteers were analyzed. The 95% limits of agreement ranged from −0.03 to 0.03 mm, −0.08 to 0.07 mm, −0.18 to 0.18 diopters (D), −1.09 to 1.16 D, −1.18 to 1.15 D for axial length (AL), anterior chamber depth (ACD), mean keratometry, J0 and J45 respectively, which were all comparable between the two biometers, while significant differences were detected in lens thickness (LT), central corneal thickness (CCT), white-to-white (WTW) and pupil diameter (PD). Predicted intraocular lens (IOL) powers were comparable between the two biometers by Haigis and Barrett Universal II formulas, while not by SRK/T, Hoffer Q and Holladay 2. Excepting CCT, WTW and PD meaurements, IOLMaster 700 and OA-2000 have excellent agreement on ocular biometric measurements and astigmatism power vectors, which provides more options for ocular biometric measurements and enables constant optimization for IOL power calculation.

## Introduction

Ocular biometric measurements have been shown to be crucial in many ophthalmic studies and clinical practices. They are often used in the calculation of intraocular lens (IOL) power, the screening of refractive surgery candidates, the diagnosis of primary angle-closure glaucoma and the monitoring of ametropic progression. Among different technologies for ocular biometric measurement, optical biometry has been proven to be more accurate and safer than ultrasonic biometry^1,2^, which is likely accompanied by the risk of infection and indentation due to contact measurement. As a result, optical biometry has increasingly gained popularity and related devices have been gradually introduced, such as IOLMaster 500 (Carl Zeiss Meditec, Jena, Germany) based on partial coherent interferometry (PCI), Lenstar LS 900 (Haag Streit AG, Koeniz, Switzerland) based on optical low-coherent reflection and Aladdin (Topcon, Tokyo, Japan) based on optical low-coherent interference, and so on. New technologies and instruments for accurate measurement of ocular biometric parameters are still needed to meet growing expectations.

In recent years, novel non-contact and high-resolution optical biometric devices, such as IOLMaster 700 (Carl Zeiss Meditec AG, Jena, Germany) and the OA-2000 (Tomey, Nagoya, Japan), have been available. Both biometers are based on swept-source optical coherence tomography (SS-OCT), the newest variations of Fourier-domain optical coherence tomography (OCT)^3^, in which the interference patterns go through a process known as Fourier transformation allowing all light echoes to be measured simultaneously. The IOLMaster 700 combines SS-OCT with a tunable laser wavelength centering on 1,055 nm (a wavelength varying from 1,035 to 1,095 nm) and a multidot keratometer. This device performs optical B-scans (optical cross-section) for measuring the ocular parameters, allowing the visualization of axial anatomical structures as a two-dimensional OCT image and ensuring fine alignment by the presence of the fovea. The OA-2000 incorporates SS-OCT and a Placido-disc topographer. The Fourier domain optical interference is utilized to measure the ocular parameters using a 1060 nm infrared light. A search function of B-scan automatically finds a measurable point for ocular parameters. SS-OCT with a longer wavelength can perform scans faster and yield higher resolution than the previous OCT^4,5^.

Agreement between the SS-OCT-based IOLMaster 700 or OA-2000 and a standard PCI biometer−IOLMaster 500 was demonstrated by previous studies^6,7^. However, to the best of our knowledge, few studies have been reported so far on the comparison of ocular biometric parameters between the new commercially available SS-OCT versions of the IOLMaster 700 and OA-2000. Therefore, the present study aims to provide a clinical reference by analyzing the measurements and evaluating the agreements of not only the axial length (AL) but the anterior segment parameters, including anterior chamber depth (ACD), central corneal thickness (CCT) and lens thickness (LT), keratometry readings (K), white-to-white (WTW) distance, pupil diameters (PD), corneal astigmatism of Cartesian (J0) and oblique (J45) components by power vector analyses, and IOL power calculation between IOLMaster 700 and OA-2000 in healthy eyes for the first time.

## Results

One hundred and three eyes of 103 subjects (57 females and 46 males), with a mean age of 23 ± 5.4 years (range 15 to 35 years) were recruited for this study. The mean spherical equivalent refraction was −1.50 ± 1.10 diopters (D) (range 0 to −3.00 D). Table 1 describes and compares the ocular biometric measurements and power vectors of corneal astigmatism from the two SS-OCT-based devices, the IOLMaster 700 and OA-2000. Table 2 showed the comparisons of IOL power calculation between the two biometers using various formnulas.

**Table 1:** The differences in biometric measurements between the IOLMaster 700 and OA-2000.

Parameter	IOLMaster 700	OA-2000	Mean difference	*P* value	95% LoA
AL (mm)	24.08 ± 0.95	24.08 ± 0.95	0.00 ± 0.02	0.051	−0.03~0.03
ACD (mm)	3.57 ± 0.26	3.57 ± 0.26	0.00 ± 0.04	0.260	−0.08~0.07
K_m_ (D)	43.61 ± 1.49	43.61 ± 1.50	0.00 ± 0.09	0.897	−0.18~0.18
K_f_ (D)	43.12 ± 1.43	43.07 ± 1.45	0.04 ± 0.11	<0.001	−0.17~0.25
K_s_ (D)	44.13 ± 1.58	44.14 ± 1.59	−0.01 ± 0.16	0.500	−0.32~0.30
LT (mm)	3.62 ± 0.20	3.70 ± 0.20	−0.08 ± 0.04	<0.001	−0.16~0.01
CCT (µm)	546.77 ± 33.19	529.69 ± 31.67	17.08 ± 3.87	<0.001	9.49~24.67
WTW (mm)	12.01 ± 0.40	11.87 ± 0.53	0.14 ± 0.34	<0.001	−0.53~0.81
PD (mm)	4.72 ± 0.86	6.18 ± 0.88	−1.46 ± 0.79	<0.001	−3.01~0.10
J0 (D)	0.06 ±± 0.40	0.03 ± 0.41	0.04 ± 0.57	0.511	−1.09~1.16
J45 (D)	0.00 ± 0.41	0.01 ± 0.44	−0.01 ± 0.59	0.819	−1.18~1.15

AL: Axial length; ACD: anterior chamber depth; LT: Lens thickness; K_m_: mean keratometry; K_f_: the flattest keratometry; K_s_: the steepest keratometry; J0: Jackson cross-cylinder, axes at 0 degrees and 90 degrees; J45: Jackson cross-cylinder, axes at 45 degrees and 135 degrees; CCT: central corneal thickness; WTW: white to white distance; PD: pupil diameters; LoA: limits of agreement.

**Table 2:** The differences in IOL power calculation between the IOLMaster 700 and OA-2000.

Formulas	IOLMaster 700	OA-2000	Mean difference	*P*	95% LoA
Haigis	20.15 ± 2.21	20.15 ± 2.36	−0.01 ± 0.32	0.880	−0.64~0.62
SRK/T	19.55 ± 2.22	19.34 ± 2.11	0.21 ± 0.49	<0.001	−0.75~1.17
Hoffer Q	19.68 ± 2.37	19.14 ± 2.29	0.53 ± 0.26	<0.001	0.02~1.04
Holladay 2	19.53 ± 2.28	19.27 ± 2.18	0.26 ± 0.30	<0.001	−0.33~0.85
Barrett Universal II	19.31 ± 2.16	19.26 ± 2.15	0.05 ± 0.33	0.100	−0.60~0.70

Note: the unit of IOL power is expressed in diapters.

### Differences in parameters measured by IOLMaster 700 and OA-2000

As shown in Table 1, two instruments provided comparable AL, ACD, steep K (K_s_) and mean K (K_m_) measurements (*P* = 0.051, 0.260, 0.897 and 0.500, respectively), whereas the difference of measured flat K (K_f_) was statistically significant (*P* < 0.001). The LT, CCT, WTW and PD values also showed statistically significant differences (*P* < 0.001). The differences in the J0 and J45 vector components of corneal astigmatism between the two biometers were similar (*P* = 0.511 and 0.819, respectively). With respect to IOL power calculation, the two biometers provided similar IOL power predictions when using Haigis and Barrett Universal II formulas (*P* = 0.880 and 0.100, respectively), whereas significant differences were shown when using SRK/T, Hoffer Q and Holladay 2 (*P* < 0.001).

### Agreement of measurements between IOLMaster 700 and OA-2000

**Figure 1:**
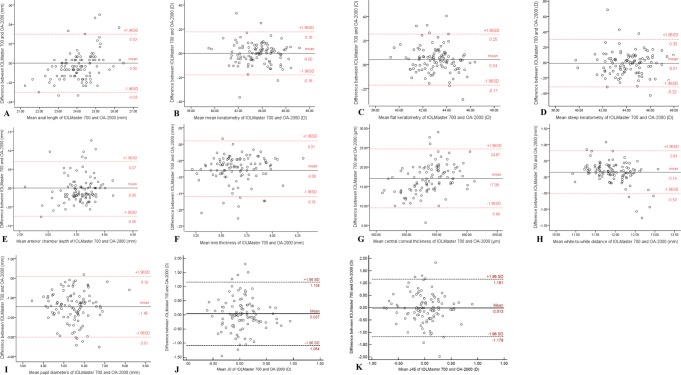
Bland-Altman plots for the AL (**A**), Km (**B**), Kf (**C**), Ks (**D**), ACD (**E**), LT (**F**), CCT (**G**), WTW (**H**), PD (**I**), J0 (**J**) and J45 (**K**) measurements with IOLMaster 700 and OA-2000.

Figure 1 demonstrates the Bland-Altman plots for the assessment of agreement of various biometric parameters between the two SS-OCT devices. As shown in Table 2, the measurements of AL, K_m_, K_f_, K_s_, ACD, LT, J0 and J45 showed better agreement than those of other parameters with relatively narrow 95% LoA. Thereinto, the agreement of AL was excellent with the narrowest 95% LoA (range −0.03 to 0.03 mm). The 95% LoA of corneal topography measurements including K_m_, K_f_ and K_s_ were in a narrow range with maximum value of −0.32 to 0.30 D, although K_f_ of them showed significant differences (*P* < 0.001). However, the measurements of CCT, WTW and PD showed poor agreement with 95% LoA (range 9.49 to 24.67 mm, −0.53 to 0.81 mm and −3.01 to 0.10 mm, respectively). For IOL power calculation, the two biometers demonstrated excellent agreement with narrow 95% LoA when using Haigis (range −0.64 to 0.62 D) and Barrett Universal II (range −0.60 to 0.70 D) formulas, which were significantly better than SRK/T (range −0.75 to 1.17 D), Hoffer Q (range 0.02 to 1.04 D) and Holladay 2 (range −0.33 to 0.85 D).

## Discussion

Technology for ocular biometric measurement continues to evolve. Recently, the optical biometric instruments IOLMaster 700 and OA-2000 have been applied in clinical practice, and a few studies have been conducted on these new instruments based on SS-OCT. Although it has been reported that either of instruments provides repeatable measurements with a deeper imaging range, less sensitivity reduction and faster scanning speeds^8–11^, there were no studies comparing the agreement between IOLMaster 700 and OA-2000 on ocular biometric parameters in subjects with transparent lens to date. We therefore performed an agreement analysis between the IOLMaster 700 and OA-2000 based on the definitions adopted by Bland and Altman recommended by the British standards body^12^.

In the present study, IOLMaster 700 and OA-2000 offered close measurements, and most of biometric parameters showed good agreement, especially for the AL and ACD values. As most key parameters for calculating and predicting IOL power, AL and ACD error attributes to 54% and 38% of the refractive error, respectively^13^. Our results showed minor difference between both biometers for measuring AL and ACD, with a mean difference of 0.002 mm and 0.004 mm, respectively (*P* = 0.051). Given that a 0.1 mm AL error is equivalent to a refractive error of about 0.27 D on the plane spectacle^14^, the difference by only 0.002 mm was also clinically insignificant. Likewise, the maximum 95% LoA of the AL and ACD with 0.03 mm and 0.08 mm between these two biometers were considered to be clinically acceptable. Kongsap *et al*.^6^ reported that the average difference in AL and ACD between OA-2000 and IOLMaster 500 in cataract patients were 0.06 mm and 0.21 mm respectively, which was statistically significant. Hua *et al*.^10^ also indicated that the AL and ACD values measured by Tomey OA-2000 and IOLMaster 500 were comparable in healthy eyes, with a mean difference of 0.058 ± 0.094 mm and 0.010 ± 0.075 mm (*P* < 0.05) and the 95% LoA no more than 0.24 mm and 0.14 mm, respectively. These differences between OA-2000 and IOLMaster 500 (PCI biometer) were more than our differences between OA-2000 and IOLMaster 700, suggesting that better agreement between the two SS-OCT in this study. The reason for this is likely due to the fact that both OA-2000 and IOLMaster 700 provide fixation monitoring function, making the measurement more controllable and repeatable. More specifically, IOLMaster 700 provides a visualization image along the longitudinal section to gain the foveal fixation, while OA-2000 provides an automatic tracing to ensure the ocular alignment fixation.

The LT was the most variable parameter due to ocular accommodation. Ferrer-Blasco *et al*.^15^ investigated the effect of accommodation on LT using IOLMaster 700, and revealed a statistically significant increase of 30 μm in LT between the subject looking at the stimulus of the biometer and the outside target at 0D of vergence. According to our data, the mean LT difference between the two devices was 0.075 mm, which was statistically significant (*P* < 0.001). The difference could be explained by the distinction in working distance and sighting targets between IOLMaster 700 and OA-2000, and the difference was even more significant among the young subjects with well accommodation. Our results were in good accordance with those previously reported between the OA-2000 and Lenstar^16^ and between the Lenstar and IOLMaster 700^17^. Although LT was not used as a variable in the calculation of IOL power previously, the fourth-generation formula such as Olsen has taken this into account^18^. Therefore, the impact of LT difference on the refractive prediction error of pseudophakic eye may not be negligible in the new-generation formulas for IOL power calculation, and further studies are needed.

The mean difference values of K_m_, K_f_ and K_s_ measured in our study were 0.00 ± 0.09 D, 0.04 ± 0.11 D and −0.01 ± 0.16 D, respectively, among which the difference in K_f_ was statistically significant (*P* < 0.001). In this study all the K_f_ measured by IOLMaster 700 were significantly higher than those measured by OA-2000. Previous study suggested that a difference of 1.0 D in keratometric value would cause a difference of about 1.40 D in the calculation of IOL power^10^. Accordingly, it could be inferred that the difference of 0.04 D in keratometric value would lead to a difference of about 0.06 D in IOL power calculation, which was far less than the increment of the IOL power step of 0.50 D. Meanwhile, the mean differences in J0 and J45 vector components of corneal astigmatism between the two biometers were 0.04 ± 0.57 D and −0.01 ± 0.59 D respectively, which were considered clinically negligible. These results were also in accordance with the study by Sabatino *et al*.^19^ that compared IOLMaster 700 with a new SS-OCT biometer Argos (Movu, Aichi, Japan), which showed a mean difference of −0.01 D in J0 and 0.05 D in J45. However, the 95% LoA of J0 and J45 in the present study (−1.09 to 1.16and −1.18 to 1.15, respectively) were remarkably larger than that in Sabatino *et al*.’s study (−0.46 to 0.44 and −0.26 to 0.36, respectively). These findings might be due to the different technologies of the two SS-OCT devices. Although both data were collected by projecting light into the central zones on the corneal surface, the IOLMaster 700 obtains K values in 18 reflected spots in hexagonal patterns at 3 zones (1.5 mm, 2.5 mm and 3.5 mm) by a distance-independent telecentric keratometer, while the OA-2000 provides K readings in concentric circles at 3 zones (2 mm, 2.5 mm and 3.0 mm) by Placido-based corneal topographer.

The CCT value in the current study also demonstrated significant differences between the two biometers *(P* < 0.001), and the average value measured by OA-2000 was 17.08 μm less than that by IOLMaster 700. This difference may also not be ignored in the new generation of prediction formulas like Olsen that includes this variable. In addition, it may affect the measurement of intraocular pressure. A study by Kohlhaas *et al*.^20^ indicated an approximately 1 mmHg correction for every 25 μm deviation from a CCT of 550 μm. Also, the CCT measurement plays a key role in the preoperative evaluation of keratorefractive surgery to avoid postoperative corneal ectasia^21^. For the WTW and PD measurements, the differences were significant and the range of 95% LoA was clinically wide. The differences could be related to the variations in the light source for image acquisition and the algorithms for edge detection around the iris between the two biometers^22^. Given that WTW measurement is required in the IOL formulas like Holladay 2, the power calculation between the two biometers may thus be varying.

It has been evolving in IOL power calculation formulas in the pursuit of optimal postoperative visual quality. As the third-generation formulas, SRK/T and Hoffer Q have inclued AL, K readings and A-constant for calculation. In the present study, the mean differences in IOL power measured by the two biometers were 0.21 D and 0.53 D respectively for SRK/T and Hoffer Q, with poor agreement indicated by large maximum 95% LoA values of 1.17 D and 1.04 D respectively. As the fourth-generation formulas, Haigis has involved AL and ACD as key parameters, while Holladay 2 has employed AL, K readings, ACD, LT and WTW for calculation. In the present study, the mean differences in measured IOL power by the two biometers were −0.01 D for Haigis while 0.26 D Holladay 2, with a maximum 95% LoA value of −0.64 D for Haigis while 0.85 D for Holladay 2. As the latest formula, excellent agreement by the two biometers was demonstrated in Barrett Universal II formula in the present study, this might due to the excellent agreement found in the AL and ACD measurements as discussed above. The findings by the present study that Haigis and Barrett Universal II formulas provided the most reliable and accurate IOL power prediction was also supported by other studies^23–26^.

The present study is limited by the fact that we only recruited healthy volunteers with transparent refractive media, whereas some of the potentially advantageous features of the SS-OCT were not fully revealed. For example, previous studies have shown that SS-OCT biometer improved tissue penetration and success rate in patients with opaque media or dense cataract^27^. While the performance of two SS-OCT biometers in the calculation and prediction of IOL power was not evaluated for cataract patients, this would be a subject for future research. In addition, the AL of our subjects ranged from 21.25 to 26.65 mm, so our results may not be suitable for those cases with axial length beyond this range, especially hypermyopia and hyperpresbyopia.

In conclusion, the two SS-OCT-based biometers provided similar measurements of the main biometric parameters, and the AL values demonstrated the best agreement among the available parameters. However, the agreement is not perfect and given some differences, the instruments cannot be deemed fully interchangeable.

## Methods

### Study design and participants

This prospective cross-sectional study included consecutive young subjects with healthy eyes from Affiliated Hospital of North Sichuan Medical College in July and August 2018. All procedures adhered to the tenets of Helsinki Declaration and the research protocol was approved by the Institutional Review Board of Affiliated Hospital of North Sichuan Medical College [2018ER(A)020]. Informed consents were obtained from all participants after the nature and possible consequences of the study were explained to them. Given that the statistical methods for agreement studies recommended the sample size at least 100 subjects, no sample size calculation was performed^28^.

Inclusion criteria included healthy subjects with a distance corrected visual acuity equal to or better than 20/20 in each eye, without a recent history of wearing contact lenses (soft lenses within 2 weeks or rigid lenses within 4 weeks) or dry eye. All subjects could communicate well and cooperate with good visual fixation. Exclusive criteria were any ocular pathology or systemic disease with ocular complications. The previous history of intraocular and corneal surgery or ocular trauma was also excluded. Only the right eyes were included for the outcome measurements.

### Measurement protocol and instruments

Routine examinations included uncorrected and corrected distance visual acuity, refraction, non-contact tonometry, slit-lamp and ophthalmoscopy. For the IOLMaster 700 and OA-2000 optical biometers, the mean values of all axial measurements, including AL, ACD, CCT and LT were acquired. Keratometric values, including K_f_, K_s_ and K_m_, were derived from the anterior corneal curvature. Based on the above keratometric values, power vector analyses of J0 (Jackson cross-cylinder, axes at 0 degrees and 90 degrees) and J45 (Jackson cross-cylinder, axes at 45 degrees and 135 degrees) components of corneal astigmatism was performed following the method by Thibos *et al*.^29^. WTW and PD were also recorded according to the collected images. The IOL power calculation data were also acquired for the AcrySof SN60WF IOL (Alcon Laboratories, Inc., Fort Worth, TX, USA). Formulas employed in both biometers were used for IOL power comparison. The order in which the biometers were employed was randomized. Subjects were asked to keep both eyes open and focus on one target during each scan, after blinking completely to allow the tear film to spread over the cornea^30^. Each of parameters per instrument was measured at least three times by a single well-trained examiner in a dimly lit room. All measurements were done within the shortest time possible, between 9:00AM and 5:00PM. The quality control criteria for both devices were implemented in line with the manufacturers’ recommendations. Any measurement with borderline signal quality shown in the quality test was repeated.

### Statistical analysis

Statistical analysis was conducted using the software SPSS 23.0 (SPSS Inc., Chicago, IL, USA). Data normality was estimated with the Kolmogorov-Smirnov test. Normally distributed data were compared by the paired *t* test. The agreement was assessed using the Bland-Altman plots^12^, which indicates the mean of measurements (x-axis) against their differences between the two SS-OCT instruments (y-axis). The 95% limits of agreement (LoA) were defined as the mean difference ±1.96 standard deviation (SD). A narrower 95% LoA indicated better agreement between measurements. A *P* value less than 0.05 was considered to denote statistical significance.

## Data Availability

The datasets generated during and/or analyzed during the current study are available from the corresponding author on reasonable request.
